# The Experience of Oncology Healthcare Providers in the Central Italy during the COVID-19 Lockdown

**DOI:** 10.3390/cancers12103031

**Published:** 2020-10-18

**Authors:** Alessandra Fabi, Patrizia Pugliese, Pina Tiziana Falbo, Domenico Corsi, Maria Agnese Fabbri, Bruno Vincenzi, Emilio Bria, Francesco Angelini, Alessandro Bonucci, Arianna Pellegrino, Chiara Falcicchio, Anita Caruso, Luca Giacomelli, Valentina Mirisola, Simonetta Papa, Francesco Cognetti, Gennaro Ciliberto, Maria Perrone

**Affiliations:** 1 Phase I Studies and Precision Medicine Unit, IRCCS Regina Elena National Cancer Institute, 00144 Rome, Italy; 2 Psyconcology Unit, IRCCS Regina Elena National Cancer Institute, 00144 Rome, Italy; patrizia.pugliese@ifo.gov.it (P.P.); alessandro.bonucci@ifo.gov.it (A.B.); chiara.falcicchio@ifo.gov.it (C.F.); maria.perrone@ifo.gov.it (M.P.); 3 Istituto Neurotraumatologico Italiano (INI), Grottaferrata, 00046 Rome, Italy; tiziana.falbo@gruppoini.it; 4 Medical Oncology, San Giovanni Calibita Fatebenefratelli Hospital, 00100 Rome, Italy; domenico.corsi@libero.it; 5 Medical Oncology, Brelcolle Hospital, 01100 Viterbo, Italy; agnese.fabbri@yahoo.it; 6 Medical Oncology, University Campus Biomedico, 00100 Rome, Italy; b.vincenzi@unicampus.it; 7 Comprehensive Cancer Center, Fondazione Policlinico Universitario Agostino Gemelli IRCCS, Università Cattolica del Sacro Cuore, 00100 Rome, Italy; emilio.bria@policlinicogemelli.it; 8 Medical Oncology, Regina Apostolorum Hospital, Albano Laziale, 00041 Rome, Italy; angelini.f@libero.it; 9 Medical Oncology, G. Paolo II P.O. Vito Fazzi, 73100 Lecce, Italy; arianna_pellegrino@libero.it; 10 Psiconcology Service, IRCCS Regina Elena National Cancer Institute, 00144 Rome, Italy; anita.caruso@ifo.gov.it; 11 Polistudium SRL, 20135 Milan, Italy; luca.giacomelli@polistudium.it (L.G.); valentina.mirisola1@posta.istruzione.it (V.M.); simonetta.papa@polistudium.it (S.P.); 12 Medical Oncology 1, IRCCS Regina Elena National Cancer Institute, 00144 Rome, Italy; francesco.cognetti@ifo.gov.it; 13 Department of Clinical and Molecolar Medicine, Sapienza University, 00100 Rome, Italy; 14 Scientific Direction, IRCCS Regina Elena National Cancer Institute, 00144 Rome, Italy; gennaro.ciliberto@ifo.gov.it

**Keywords:** COVID-19, survey, emotional distress, healthcare providers

## Abstract

**Simple Summary:**

Few data in the literature are available about the psychological impact of COVID-19 pandemic on healthcare providers in Italy, especially with regards to different regions. In the present work, the “VIRARE” survey was addressed to all the healthcare providers in the Lazio region and, in particular, to those working in the oncology field. Healthcare providers’ opinions on the impact and on the management of the pandemic have been analyzed, to provide an exhaustive overview about the level of their experienced psychological distress.

**Abstract:**

While the emotional response of healthcare providers during the COVID-19 pandemic has been extensively investigated in countries in the Far-East, little is known about the psychological impact and the associated emotional distress of healthcare providers in Italy, especially with regard to different regions. The aim of the “VIRARE” survey, which was addressed to all the healthcare providers in the Lazio region (central Italy) and, in particular, to those working in the oncology field, is to analyze their opinion on the impact and management of the pandemic, to better understand the level of their psychological distress. A global good psychological response of healthcare providers to the pandemic has emerged, independently from their different occupations in the oncology field. Healthcare providers show a high degree of resilience, identifying the major causes of distress the difficulty of the management of this situation, the obstacles in their working activity and expressing a high degree of dissatisfaction with how Italian institutions handled this situation. This survey also provides a direct comparison between COVID-19-infected (or directly in contact with COVID-19-infected patients) and uninfected healthcare providers, identifying the sub-category of infected professionals that reported signs of depression as particularly vulnerable.

## 1. Introduction

The coronavirus disease 2019 (COVID-19) pandemic remains a public health emergency of international concern [1]. Italy was the first Western country to face the pandemic. Unfortunately, the Italian regional healthcare system was overwhelmed by the impossibility of effectively responding to the needs of the multitude of patients [2], and the implementation of measures to protect patients and healthcare providers from COVID-19 infection [3,4,5]. Up to May 2020, a record 21,981 healthcare providers were infected and more than 200 of them died in Italy due to COVID-19 [6]. 

This severe emergency consequently caused major distress to healthcare providers, mainly due to the lack of organization to counteract this unexpected emergency (e.g., shortage of personal protective equipment). In addition, they were subjected to emotional distress caused by the fear of getting infected and the fear of infecting close relatives (patients, family and friends), and this feeling was further worsened by the perception of the absence of an immediate remedy for this problem. 

While the emotional distress of healthcare providers exposed to SARS-CoV-2 infection has already been investigated in different countries, especially in countries in the Far-East [7,8,9,10,11], few data in the literature are available about the psychological impact of the COVID-19 pandemic and the associated emotional distress of healthcare providers in Italy [12,13]. In particular, considering that the pandemic had a different impact in different areas of Italy, there is a lack of information from single regions. Furthermore, to our knowledge, the emotional distress perceived by healthcare providers has never been correlated with the actual exposure to SARS-CoV-2 infection.

The “VIRARE” survey was addressed to all the healthcare providers in the Lazio region (central Italy), and in particular to those working in the oncology field. Indeed, cancer patients constitute the most relevant example of vulnerability during this COVID-19 emergency. Consequently, health operators in this field must provide a strong and efficient response to the needs of cancer patients during the pandemic, with a consequent emotional and work overload which has not been evaluated in the literature. The aim is to analyze their opinion on the impact of the pandemic and its management, and to better understand the level of psychological impact, anxiety, depression and stress. The answers to the “VIRARE” questionnaire may help identify the principal factors of emotional distress, also providing a direct comparison between COVID-19-infected (or those directly in contact with COVID-19-infected patients) and uninfected healthcare providers, between healthcare providers in direct contact with patients compared to other healthcare professionals and by stratifying healthcare providers by their field of practice and place of work.

## 2. Results

### 2.1. Survey Respondents

In total, 472 healthcare providers in the Lazio region completed the “VIRARE” survey. A total of 82% (*n* = 387) of respondents were working in the oncology field. Among all healthcare providers, a total of 166 (39%) were tested for COVID-19 by swab or serum test. The sociodemographic and occupational characteristics of healthcare providers are summarized in Table 1.

### 2.2. Evaluation of the Impact of the COVID-19 Pandemic on the Overall Sample

In total, 50% (*n* = 212) of healthcare providers consider the measures adopted by their hospital or clinic to limit the spread of coronavirus as “quite satisfactory”. 

Most of them (*n* = 366; 86%) believed that this emergency interfered a lot in their working life. In 38% (*n* = 159) of cases, their activity was partially suspended; 20% (*n* = 87) and 14% (*n* = 58) of healthcare providers reported a decreased or unaltered workload, respectively, while 28% of cases (*n* = 121) considered their workload augmented.

Work-related stress is a potential cause of concern for healthcare professionals. Indeed, the majority of healthcare providers considered this period as quite (*n* = 291; 51%) or very (*n* = 163; 38%) distressing. Despite this perception, which was accompanied, in most cases, by a feeling of intense (*n* = 177; 42%) or very intense (*n* = 59; 14%) fear, there was no evidence for relevant psychological discomfort. Most healthcare providers did not lose their personal interests (*n* = 210; 49%), did not try to remove this event (*n* = 275; 65%) or avoided thinking about it (*n* = 205; 48%), did not have physical reactions of anxiety or stress thinking about this event (*n* = 313; 74%), and did not have sleep disturbances (*n* = 258; 60%), nor did they dream about this situation (*n* = 313; 74%).

The close proximity to a high number of COVID-19 patients, or the emergency situation experienced in a less direct way, denoted a change in the reported perception of social relations: the majority of healthcare providers (*n* = 313; 74%) claimed to feel closer to people who suffer from their health conditions, without relating this perception to an augmented perception of their religious faith (in 59% of cases, *n* = 251).

A general agreement about one’s own personal ability to deal with this emergency was reported: 79% (*n* = 335) of healthcare providers believed they dealt with this moment well, with courage (*n* = 335; 79%) and in the most correct way (*n* = 360; 84%).

### 2.3. Impact of the Pandemic on Healthcare Providers Reporting Psychological Stress

**Figure 1:**
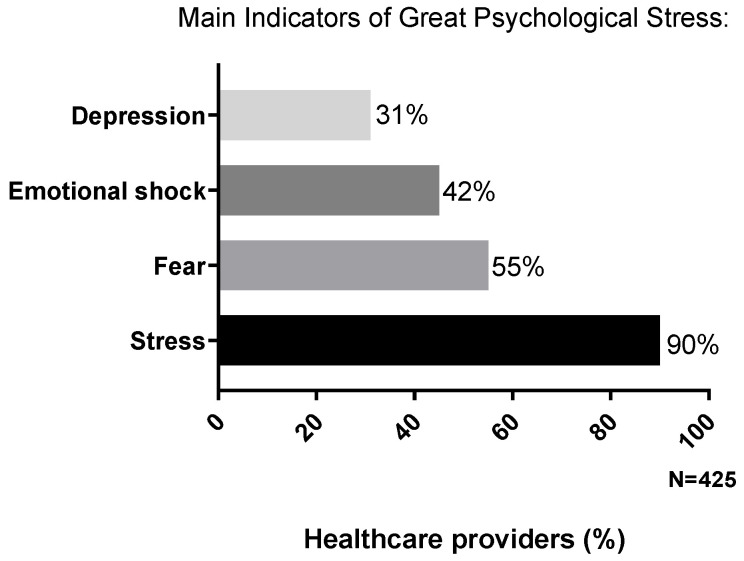
Percentage of healthcare providers who reported greater psychological distress compared to the overall population. This was categorized on the basis of four main indicators (depression feelings, emotional shock perception, sensation of fear or stress).

A further analysis on the psychological impact of this emergency was carried out by identifying four subgroups, considering only healthcare providers who answered “quite/a lot” to the following questions, denoting a greater level of psychological stress (Figure 1):

- Do you find this moment stressful? (*n* = 382; 90%);
- Do you feel sensations of fear? (*n* = 236; 55%);
- Do you consider this moment as an emotional shock? (*n* = 191; 45%);
- Do you feel depressed? (*n* = 130; 31%).

Of note, the feeling of a “stressful moment” is the most shared by healthcare providers if compared to the other indicators.

In these identified subgroups, characterized by a perception of stress, fear and shock feelings were amplified compared to the overall population, the emergency is linked in particular to the fear of terrible events (up to 76% of cases) and, above all, to a feeling of “paralysis” in their daily life (up to 94% of cases).

These categories of healthcare providers believe that all of these feelings could lead to a change in the personal scale of values (up to 74% of cases).

However, also in these subgroups, a particular degree of psychological discomfort is not reported. The event is not considered to cause physical stress reactions or sleep disturbances and also these categories of healthcare providers did not try to remove this event or avoided thinking about it, in line with what was reported by the overall population.

### 2.4. Impact of the Pandemic on Healthcare Providers Affected or Working Close to COVID-19-Infected Patients

**Figure 2:**
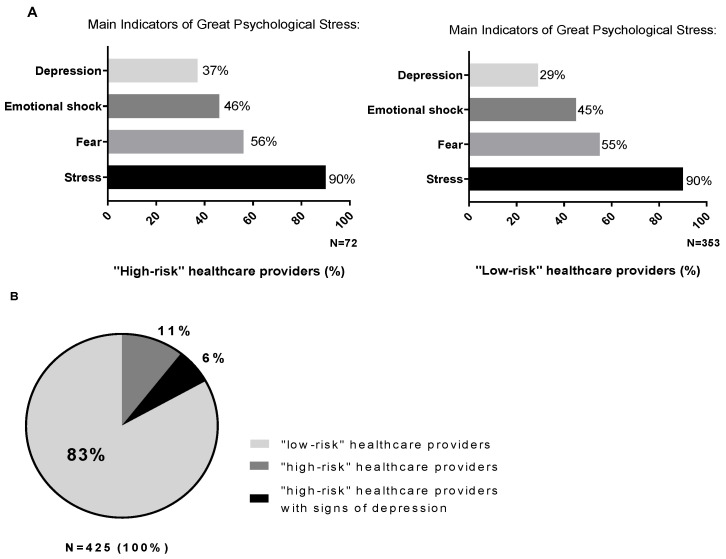
(**A**) Percentages of high- and low-risk healthcare providers who reported greater psychological distress, categorized on the basis of four main indicators (depression feelings, emotional shock perception, sensation of fear or stress). (**B**) In total, 17% of the healthcare providers worked in a COVID-19 department or contracted COVID-19 (high-risk category, dark gray and black segments). Among them, 6% reported signs of depressing feelings, linked to a worse global psychological outcome (black segment). Light gray segment corresponds to “low-risk” healthcare providers.

A total of 72 healthcare providers (17%) reported to have worked in a COVID-19 department or to have contracted COVID-19. This subgroup has been defined as healthcare providers at “high risk” of stress and psychological consequences because most healthcare professionals working in isolation units and hospitals very often did not receive any training for providing themselves mental healthcare [14]. Their answers have been analyzed and compared with those of other healthcare providers who were defined as “low risk” (*n* = 353; 83%) and who neither worked in a COVID-19 department nor contracted the infection (Figure 2B).

Among the “high-risk” healthcare providers, 56 (78%) were tested for COVID-19 and 20 (28%) received a positive diagnosis of COVID-19, resulting in home isolation. A total of 110 (31%) of healthcare providers from the “low-risk” category was tested for COVID-19 and no one from the group reported having contracted the infection.

In the high-risk population, an increased workload was reported in a higher proportion of cases (*n* = 34; 47%) compared with the low-risk population (*n* = 87; 25%), and this emergency was considered a major obstacle for the work activity for almost all high-risk healthcare providers (*n* = 67; 93%).

Even in these subgroups, the distress and the feeling of a life blockage were mainly reported. Sixty-five (90%) of the high-risk healthcare providers reported to be quite or very stressed and this opinion was also reported in the same proportion by low-risk healthcare providers.

However, the majority of high- and low-risk healthcare providers believed themselves to have handled the emergency with courage and in the most correct way.

As for the overall population, a further analysis on the psychological impact of this emergency was carried out by identifying four subgroups within the high- and low-risk sample, according to the same criteria reported above (Figure 2A). 

Among these subgroups, the presence of depressing feelings seems to have the most relevant role on the psychological outcome. 

In particular, in the high-risk healthcare providers group, 27 cases (37% of the high-risk category, 6% of the total) reported feeling depressed (Figure 2B) and the presence of psychological discomfort was underlined by sleep disturbances (*n* = 21; 78%), fear of terrible events (*n* = 26; 96%), physical reactions of stress (*n* = 12; 44%) and a feeling of oppression (*n* = 17; 63%) reported in a greater proportion.

### 2.5. Evaluation of the Impact of the COVID-19 Pandemic among Healthcare Providers According to Their Different Fields of Practice or Place of Work

The responses of health workers of the oncology field (*n* = 387; 82%) were compared to those of health workers of other settings (*n* = 85; 18%). No differences in the personal perception of the emergency and in the management of this situation emerged, compared with the overall analysis. 

The same results were obtained by further stratifying the health workers according to their profession or place of work, as reported in detail in the “occupation” and “workplace” sections of Table 1.

This suggests a common perspective about how to handle the emergency, independently of personal job experiences and skill.

### 2.6. Impact of the Pandemic on Healthcare Providers in Direct Contact with Patients Compared to Other Healthcare Professionals 

The impact of the pandemic was compared between healthcare workers in close contact with patients (*n* = 373; 77%) and those working outside a hospital setting (*n* = 9; 23%), such as psychologists, biologists and other healthcare workers (administrative workers, data managers, pharmaceutical representatives). For both groups, the emergency was perceived as a major obstacle for their work activity (*n* = 293; 86%, for hospital operators; *n* = 73; 85%, for external operators) although for different reasons, considering that workload was reported to be suspended/decreased for the majority of operators not in contact with patients (*n* = 51; 65%). This determined an increased feeling of stress along with a feeling of “paralysis” in their daily life, reported as more perceived consequences, in line with the results of the overall sample.

For instance, in 87% (*n* = 75) of cases, healthcare workers working outside s hospital setting were not tested for COVID-19. 

### 2.7. General Opinion on the Management of the Emergency

Regarding the personal opinion on how the institutions handled this emergency, most healthcare providers reported an unsatisfactory judgment; they referred to a perception of neglect and disorganization by the institutions in 73% of cases (*n* = 309) and in 78% of cases (*n* = 333), they would have liked to do something to change the management of the emergency.

This perception was also common to all subgroups that were analyzed.

## 3. Discussion

Up to May 2020, at least the 8.3% of total COVID-19 cases in Italy were represented by healthcare providers (Italian Health Institute) [6] since the onset of outbreak in February. This poses a high emotional challenge for this category, and the psychological consequences of this emergency need to be investigated.

The aim of the survey was to evaluate the psychological impact of the COVID-19 epidemic on healthcare providers in the Lazio region of central Italy, especially on those working in the oncology field, and to correlate emotional distress with the actual exposure to SARS-CoV-2 infection, an issue poorly investigated to date. The response rate was quite high. Even if not all health professional categories (such as residential care home healthcare providers) are represented, the sample (*n* = 472 of respondents out of 598 delivered surveys) could be considered representative of the healthcare provider category in central Italy (Lazio region) and in particular of those working in the oncology field, representing 82% of responders.

The survey analysis was carried out on the overall population and considered different subgroups of healthcare providers, in particular, those who reported greater psychological distress or healthcare providers who worked in a COVID-19 department or were infected.

For each subgroup analyzed, the major cause of distress seems to be related to the difficulty in the management of this situation and the obstacles in their working activity more than the anxiety of facing the health emergency. Nevertheless, a global good psychological response to the pandemic is reported. This is related in particular to the resilience of the healthcare workers, who did not stop their activity because of the emergency, nor did they physically endure the emergency. Indeed, healthcare providers reported that they did not experience any particular psychological or physical discomfort, in line with some other studies [11,15]. The healthcare provider profession is seen as a “mission to be accomplished”; healthcare providers declare to have managed the situation with courage and in the best possible way, and this perception is common to all subgroups. 

Another common perception to all healthcare providers is a global dissatisfaction with the management of the pandemic by the institutions; it emerges clearly how healthcare providers would have liked to change the management of this emergency. A considerable cause of dissatisfaction could be due to the low number of COVID-19 tests carried out: 60% of healthcare providers were not tested. Considering that this survey was carried out at the end of the first phase of the pandemic, the plan for COVID-19 tests among healthcare professionals should have been better defined. On the contrary, health workers show greater confidence towards their hospital or clinic, which implemented specific guidelines and procedures to deal with the emergency independently in most cases. 

Among health workers of COVID-19 departments or those affected by COVID-19 (high-risk category), the sub-category that reported signs of depression was found to be particularly vulnerable. Even if they represent a small part of the sample (27 out of 72 cases), they can be considered as a category which needs an enhanced sustenance; a psychological support plan could be implemented and suggested for this category. Indeed, even if the COVID-19 emergency exalted the role of the healthcare providers among the “non-experts”, on the part of the operators themselves, there is the awareness of the impossibility of predicting catastrophic phenomena with such a high number of victims and the impossibility to cope with all aspects of public safeguards without adequate support. 

## 4. Materials and Methods 

A team of oncologists and psychologists working in the oncological setting developed the “VIRARE” survey—Il VIssuto dell’opeRAtoRE Sanitario nell’era COVID-19 (the experience of the health worker in the COVID-19 era)—at IFO Regina Elena in Rome, a referral cancer center in central Italy; the Italian Association of Medical Oncology (AIOM) and the Italian Society of Psycho-Oncology (SIPO) endorsed the survey. 

A questionnaire of 39 items covering several areas was designed: (1) demographic and occupational data (seven questions); (2) daily work organization during the epidemic (three questions); (3) personal information with respect to the management of COVID-19 infection and precautionary measures (five questions); (4) psychological impact of the COVID-19 outbreak and mental health status (twenty-two questions); and (5) opinion on the emergency management (two questions).

The survey was addressed to all the healthcare providers in the Lazio region (private or public structures), in particular to those working in the oncology field, aged ≥ 30 years and able to access the online survey anonymously delivered by the Survey Monkey™ tool (SVMK Inc., San Mateo, CA, USA), from 25 April to 3 May 2020. 

Potential participants were contacted via the mailing lists of AIOM and SIPO members or by announcements on social networks. People who wished to participate were asked to contact the AIOM scientific secretary to verify if they met the inclusion criteria.

On the basis of collected data, a general overview on the psychological impact of COVID-19 pandemic was provided, along with comparisons between different subpopulations of healthcare providers. In particular, the following comparative analyses evaluating the psychological response were carried out: overall population compared to healthcare providers who reported a greater level of psychological stress, or healthcare providers affected/working close to COVID-19-infected patients; a comparison between healthcare providers working in the oncology field and other healthcare providers; a comparison between healthcare providers stratified by their different fields of practice or places of work.

All data were analyzed by descriptive statistics by using a Microsoft Office Excel worksheet.

## 5. Conclusions

In conclusion, this survey gives a comprehensive insight into the personal experience of health workers in central Italy during the COVID-19 pandemic, suggesting an overall good psychological response to this extraordinary emergency situation. Of note, this perception is common to all the healthcare providers, independently from their field or place of work, suggesting the awareness by each healthcare professional of their mission. To better define this particular aspect, ad hoc psychological studies for health professionals would be necessary. Furthermore, even though the Lazio region had the least contagion and mortality thanks to the early organization of prevention, mainly due to the delayed onset of the first infections, this survey reveals a request for more support from the institutions.

## Figures and Tables

**Table 1:** Healthcare provider sample description.

Characteristic	*n* (%)
Gender Female Male	320 (68%) 152 (32%)
Mean age (range); years	48 (32–65)
Healthcare providers with children	331 (70%)
**Family size** Nobody 1 person 2 people 3 people >3 people	54 (11%) 90 (19%) 93 (20%) 130 (28%) 105 (22%)
Occupation General practitioner Oncologist Surgery specialist Radiotherapist Gynecologist Endocrinologist Pneumologist Radiologist Pathologist Neurologist Orthopedic Gastroenterologist Psychologist Biologist Pharmacist Other healthcare professional Nurse/social health operator	7 (1%) 77 (16%) 37 (8%) 12 (2%) 13 (3%) 10 (2%) 11 (2%) 21 (4%) 6 (1%) 4 (1%) 5 (1%) 60 (13%) 21 (4%) 31 (6%) 4 (1%) 47 (13%) 106 (22%)
Workplace Private outpatient clinic Hospital outpatient clinic Frontline department in COVID-19 patient care (first aid, intensive and sub-intensive care, etc.) Other hospital department Hospital pharmacy Extra hospital pharmacy Laboratory Other	13 (3%) 127 (27%) 30 (6%) 176 (37%) 2 (<1%) 1 (<1%) 43 (9%) 79 (17%)
